# Marine Bromophenol Derivative 3,4-Dibromo-5-(2-bromo-3,4-dihydroxy-6-isopropoxymethyl benzyl)benzene-1,2-diol Protects Hepatocytes from Lipid-Induced Cell Damage and Insulin Resistance via PTP1B Inhibition

**DOI:** 10.3390/md13074452

**Published:** 2015-07-17

**Authors:** Jiao Luo, Ning Wu, Bo Jiang, Lijun Wang, Shuaiyu Wang, Xiangqian Li, Baocheng Wang, Changhui Wang, Dayong Shi

**Affiliations:** 1 Key Laboratory of Experimental Marine Biology, Institute of Oceanology, Chinese Academy of Sciences, Qingdao 266071, China; E-Mails: luojiao2012@163.com (J.L.); wuning@qdio.ac.cn (N.W.); beckyjiang0220@163.com (B.J.); wanglijun@qdio.ac.cn (L.W.); 12-12sy@163.com (S.W.); lnu101@163.com (X.L.); wbache@163.com (B.W.); 2 The University of Chinese Academy of Sciences, Beijing 100049, China; 3 Qingdao Medical University Affiliated Hospital, Qingdao 266070, China; E-Mail: docjack@163.com

**Keywords:** HPN, palmitate, PTP1B inhibition, anti-cell damage, anti-insulin resistance, HepG2 cell

## Abstract

3,4-Dibromo-5-(2-bromo-3,4-dihydroxy-6-isopropoxymethyl benzyl)benzene-1,2-diol (HPN) is a bromophenol derivative from the marine red alga *Rhodomela confervoides*. We have previously found that HPN exerted an anti-hyperglycemic property in *db*/*db* mouse model. In the present study, we found that HPN could protect HepG2 cells against palmitate (PA)-induced cell death. Data also showed that HPN inhibited cell death mainly by blocking the cell apoptosis. Further studies demonstrated that HPN (especially at 1.0 μM) significantly restored insulin-stimulated tyrosine phosphorylation of IR and IRS1/2, and inhibited the PTP1B expression level in HepG2 cells. Furthermore, the expression of Akt was activated by HPN, and glucose uptake was significantly increased in PA-treated HepG2 cells. Our results suggest that HPN could protect hepatocytes from lipid-induced cell damage and insulin resistance via PTP1B inhibition. Thus, HPN can be considered to have potential for the development of anti-diabetic agent that could protect both hepatic cell mass and function.

## 1. Introduction

Type 2 diabetes mellitus (T2DM) is a complex metabolic disorder resulting in insulin resistance of the peripheral tissues and impaired insulin secretion from the pancreas [1,2]. Several specific molecular targets in the insulin signaling pathway have been considered as novel therapeutic approaches for type 2 diabetes [3,4]. The insulin signaling is initiated when insulin binds to insulin receptor (IR) which results in IR auto-phosphorylation of key tyrosine residues [5]. Tyrosine residues of insulin receptor substrate (IRS) are subsequently phosphorylated by the IR kinase which can further trigger downstream PI3K-Akt signaling. Activated Akt plays a pivotal role in the glucose metabolism which can activate insulin-stimulated glucose transport and regulates glycogen synthesis by GSK-3α and β inactivation [6]. Phosphorylated Akt signaling cascades also have a critical role in cell survival and apoptosis regulation. Akt promotes cell survival by inhibiting programmed cell death by phosphorylating and inactivating certain members of Bcl-2 family or caspase family, such as BAD or caspase-9 [7].

Protein tyrosine phosphatase 1B (PTP1B) is a negative regulator of insulin signaling transduction which has drawn great attention in recent T2DM therapy study [2,3,4,8,9]. PTP1B, which is localized in the cytoplasmic-face membrane of the endoplasmic reticulum, is widely expressed in insulin-sensitive tissues, such as liver, muscle and adipose tissues [10]. PTP1B can bind and dephosphorylate tyrosine residues of activated IR as well as IRS, resulting in impaired insulin signaling [11,12]. Previous studies have shown that PTP1B overexpression inhibits phosphorylation of IR and IRS-1, leading to insulin resistance. Deletion of PTP1B in liver, muscle and fat tissues enhances insulin sensitivity [13,14,15]. Furthermore, several human researches have indicated that insulin resistance is accompanied by an abnormal increase in PTP1B activity in adipose tissue and skeletal muscle [16,17]. Collectively, both *in vitro* and *in vivo* evidences validated PTP1B as an exciting target for T2DM treatment and drug development.

Although the significance of PTP1B in regulating insulin signaling has been widely reported, the role of PTP1B as a modulator of apoptosis was only reported in a few papers. It was reported that PTP1B deficiency protects hepatocyte cells against serum depletion-induced apoptosis [18]. Down-regulation of PTP1B by siRNA effectively protects cardiomyocytes against hypoxia-reoxygenation-induced apoptotic cell death [19]. Furthermore, PTP1B-null mice are more resistant to Fas-induced liver damage compared with wild type mice [20]. However, it remains unclear whether PTP1B inhibitor can attenuate HepG2 cell apoptosis.

Lipotoxicity is characterized by an excess of free fatty acids (FFA) in peripheral non-adipose tissues such as liver, muscle and pancreas, leading to apoptotic cell death and a loss of functional tissue mass, which may further result in cellular dysfunction [21,22,23,24,25]. Palmitic acid (PA) can lead to apoptosis in many kinds of cells, including pancreatic β-cells [22], cardiomyocytes [23], skeletal muscle cells [24], endothelial cells [25]. Previous studies have also shown that PA can cause insulin resistance in insulin-target tissues, both *in vitro* and *in vivo* [26,27,28,29]. A recent study highlighted that PA caused ER stress, apoptosis and insulin resistance in primary human and mouse hepatocytes [30]. In addition, many *in vitro* studies have also shown that some factors, such as PA, can up-regulate PTP1B expression in hepatic and skeletal muscle cells [31,32,33]. Accumulated evidence shows that PA is an important stimulus which contributes to the development of insulin resistance and cell dysfunction in type 2 diabetes. However, whether PTP1B inhibitors could attenuate PA-induced cell damage and insulin resistance in HepG2 cells remains to be comprehensively elucidated.

**Figure 1:**
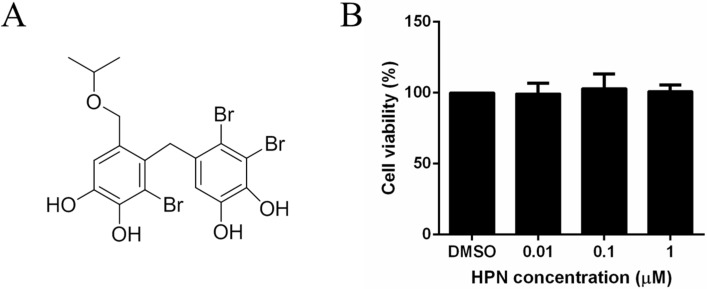
The effect of HPN on HepG2 cell proliferation. (**A**) The structure of 3,4-dibromo-5-(2-bromo-3,4-dihydroxy-6-isopropoxymethyl benzyl)benzene-1,2-diol (HPN); (**B**) The effect of HPN on cell proliferation. HepG2 cells were incubated in the presence of certain concentration or in the absence of HPN at 37 °C for 24 h, and cell viability was determined by MTT assay. All the experiments were repeated more than four times. Values represent means ± SD of quadruplicate measurements.

Marine bromophenols are a unique class of chemicals widely present in the marine algae, ascidian, and sponges, and they are reported to have diverse bioactivities including antitumor [34], antioxidant [35], anti-inflammatory [36], antifungal [37], and especially antidiabetic activities. For example, 2,4,6-tribromophenol and 2,4-dibromophenol, isolated from the red alga *Grateloupia elliptica*, effectively inhibited α-glucosidase, sucrase and maltase [38]. Bis(2,3-dibromo-4,5-dihydroxybenzyl) ether, isolated from the marine algae, is a potential α-glucosidase inhibitor for type 2 diabetes treatment [39]. In our previous study, a series of bromophenol compounds derived from the red alga *Rhodomela confervoides* have been identified as PTP1B inhibitors with anti-hyperglycemic and antidiabetic properties [40,41,42]. We have previously reported 3,4-dibromo-5-(2-bromo-3,4-dihydroxy-6-ethoxymethyl benzyl)benzene-1,2-diol (BPN) as an inhibitor of PTP1B (IC_50_ = 0.84 μmol/L). Using BPN as the initial lead compound and a structure-based strategy, we also designed and synthesized 3,4-dibromo-5-(2-bromo-3,4-dihydroxy-6-isopropoxymethyl benzyl)benzene-1,2-diol (HPN) to target PTP1B (Figure 1A). Subsequent studies have showed that HPN exhibited enhanced inhibitory activity against PTP1B (IC_50_ = 0.63 μmol/L) and specific selectivity against other members of the protein tyrosine phosphatases (PTPs) family [43]. Animal experiments with *db*/*db* mouse model demonstrated that HPN could significantly decrease plasma glucose level (*p* < 0.01) in a dose-dependent manner. However, the study of related molecular mechanisms is not enough, and many processes are still unclear.

Elevated FFA concentrations, which are common in type 2 diabetes, are linked with the onset of peripheral and hepatic insulin resistance [44]. Thus, it is of great importance to identify novel and promising agents which can reduce the effects of elevated plasma FFA in obesity and T2DM. Therefore, we report herein the effect of HPN on PA-stimulated hepatic cell damage, and the mechanism by which HPN protects hepatocytes from cell death. Furthermore, this study also explores the role of HPN in insulin resistance induced by PA and the possible molecular mechanisms underlying PA-induced cell damage and insulin resistance in HepG2 cells.

## 2. Results

### 2.1. HPN Shows No Effect on HepG2 Cell Proliferation

MTT assay was performed to test whether HPN could inhibit or promote cell proliferation of HepG2. As shown in Figure 1B, when the HepG2 cells were treated with HPN at a concentration of 1.0 μM, 0.1 μM, and 0.01 μM for 24 h, the cell viability rates were, respectively, 99.4%, 103.1%, and 98.2% when compared to the control cells. The results suggest that HPN has no significant growth-inhibiting or growth-promoting effect on HepG2 cells.

### 2.2. HPN Inhibits PA-Induced Cell Death in HepG2 Cells

**Figure 2:**
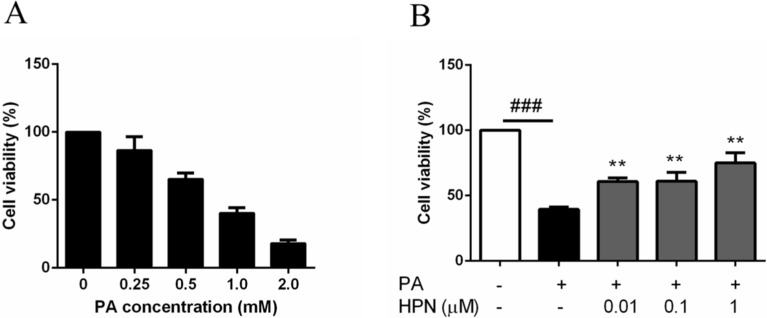
HPN blocks PA-induced hepatic cell death. (**A**) Dose response of cell death induced by PA. HepG2 cells were untreated or exposed to 0.25, 0.5, 1.0 and 2.0 mM PA for 24 h; (**B**) HPN protected HepG2 cells from PA-induced cell death. HepG2 cells were pre-incubated with HPN (1.0 μM, 0.1 μM, and 0.01 μM) for 45 min, and then stimulated with 0.5 mM PA for 32 h; ^###^ *p* < 0.001 *versus* non-treated cells, ** *p* < 0.01 *versus* PA treated cells.

The MTT assay was used to determine if HPN was able to block PA-induced cell death. As shown in Figure 2A, PA significantly decreased HepG2 cell survival rate compared to the untreated group. Cell viability rate dropped in a dose-dependent manner. When cells were treated with 0.25, 0.5, 1.0, and 2.0 mM PA for 24 h, the cell viability was decreased to 86.1%, 65.3%, 40.3%, and 17.9%, respectively.

As shown in Figure 2B, PA-induced cell death was significantly inhibited when HepG2 cells were treated with HPN. The cell viability was increased to 75.1%, 61.0%, and 60.7%, when HepG2 cells were pre-treated with 1.0 μM, 0.1 μM, and 0.01 μM HPN, compared to the PA-treated cells (39.5%). These results indicate that HPN has a significant cell death-inhibiting effect on PA-treated HepG2 cells. 

### 2.3. HPN Prevents PA-Induced Caspase-3 Activation

**Figure 3:**
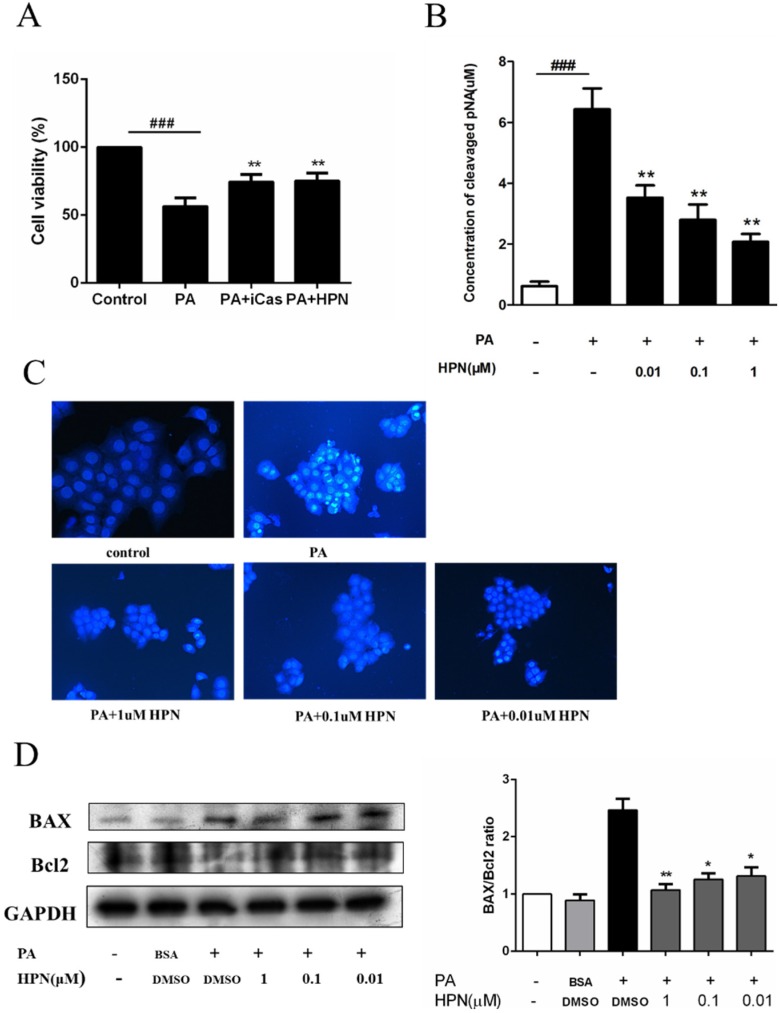
HPN prevents PA-induced caspase-3 activation. (**A**) Caspase inhibitor inhibited PA-induced cell death in HepG2. HepG2 cells were pre-incubated with HPN (1.0 μM) or caspase inhibitor, Z-VAD-FMK (20 μM), for 45 min, and then supplemented with 0.5 mM PA for 24 h. Cell survival was determined by MTT assay. ^###^ *p <* 0.001 *versus* control, ** *p <* 0.01 *versus* PA-treated group. (**B**) HPN could significantly block caspase-3 activity. After HPN and PA treatment, cells were collected and lysed on ice for 15 min, and the supernatant was taken to determine the caspase-3 activity. The absorbance at 405 nm was determined with a microplate reader and the amount of pNA was then calculated by an according standard curve; ^###^ *p* < 0.001 *versus* non-treated cells, ** *p* < 0.01 *versus* PA-treated group. (**C**) Analysis of apoptosis by Hoechst 33258 staining. HepG2 cells were pre-incubated with HPN (1.0 μM, 0.1 μM, and 0.01 μM) for 45 min, and subsequently stimulated with 0.5 mM PA for 12 h. Cells were stained with Hoechst 33258 and observed under a fluorescence microscope. (**D**) HPN improved Bcl2/BAX ratio in PA-treated HepG2 cells. HepG2 cells were pre-treated with HPN for 45 min prior to induction with 0.5 mM PA for 8 h. Immunoblotting assay was used to determine the protein levels of BAX and Bcl2. GAPDH was used as loading control; * *p* < 0.05, ** *p* < 0.01 *versus* PA-treated group.

To test whether PA-induced cell death is correlated with cell apoptosis, HepG2 cells were incubated with 0.5 mM PA in the presence of HPN or Z-VAD-FMK, a cell permeable pan caspase inhibitor. Figure 3A showed that Z-VAD-FMK could significantly attenuate PA-treated cell death. This result suggests that apoptosis was the major form of cell death induced by PA in HepG2 cells. As PA-induced cell death could also be blocked by HPN (Figure 3A), this indicates that HPN can also inhibit PA-treated cell apoptosis.

We next investigated the role of HPN on the PA-treated HepG2 cells apoptosis. HepG2 cells were incubated with 0.5 mM PA for 8 h, and then the caspase-3 activity was detected by caspase-3 activity assay kit. As shown in Figure 3B, PA caused about 10-fold increase in caspase-3 activity in comparison with the untreated cells, after 8 h of treatment. HPN pre-treatment dramatically attenuated PA-induced increase of caspase-3 activity in a dose-dependent manner. This result was also confirmed by Hoechst 33258 staining. Cell nuclear pyknosis, chromosome condensation and nuclear fragmentation were observed when HepG2 cells were treated with 0.5 mM PA for 12 h. HPN pre-treatment markedly decreased the nuclear pyknosis and karyorrhexis (Figure 3C). No apoptosis was observed in the untreated control cells. We also found that the BAX/Bcl2 expression ratio apparently decreased when cells were treated with HPN (Figure 3D).

### 2.4. HPN Ameliorates PA-Induced Insulin Resistance with Up-Regulated Insulin Signaling

**Figure 4:**
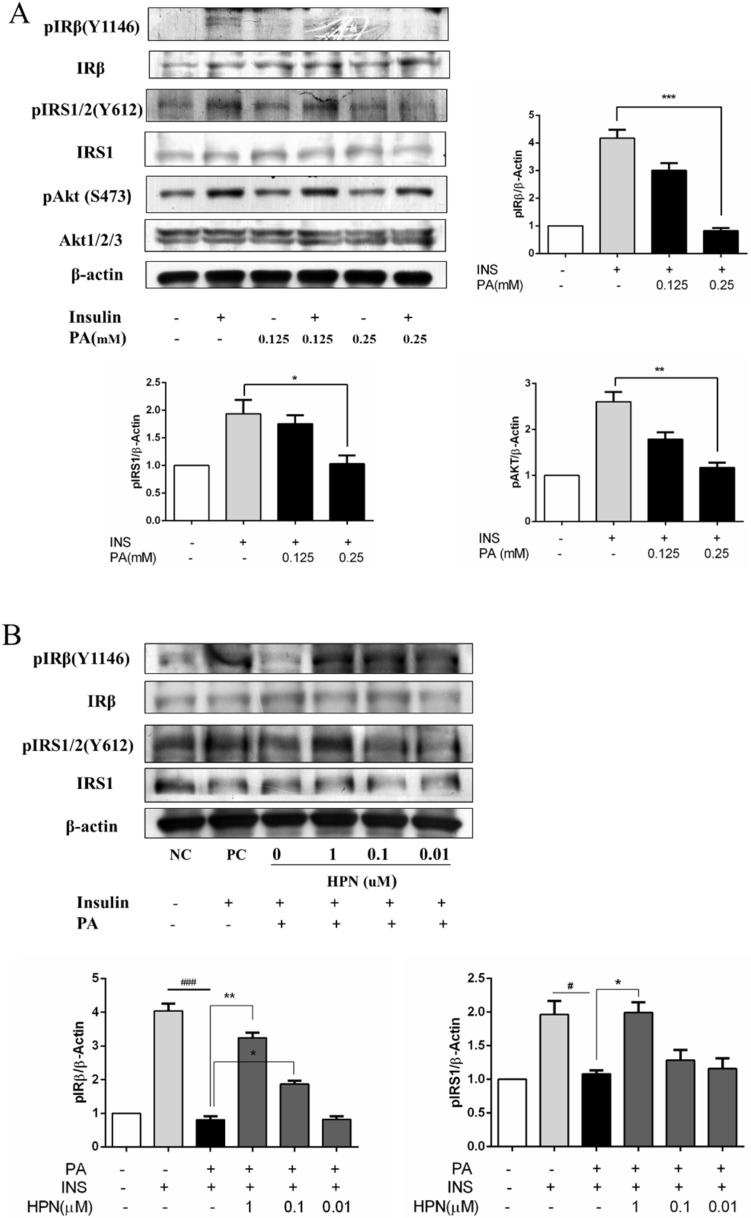
Effects of HPN on PA-induced insulin resistance. (**A**) PA inhibited insulin receptor-mediated signaling in HepG2 cells. Serum-starved HepG2 cells were untreated or treated with PA for 16 h prior to stimulation with 100 nM insulin for 30 min. Immunoblotting assay was used to determine the phosphorylated and non-phosphorylated protein levels of IRβ, IRS1/2, AKT, and GSK3α. β-Actin was used as loading control. * *p* < 0.05, ** *p* < 0.01, *** *p* < 0.001 *versus* PA-treated group. (**B**) HPN improved HepG2 cell insulin-resistance induced by PA. HepG2 cells were serum-starved with 0.5% FFA-free BSA medium, and treated with 0.25 mM PA for 16 h after HPN incubation. Subsequently, cells were stimulated with 100 nM insulin for 30 min. Western blot assay was used to detect the changes of pIRβ (Y1146), pIRS1/2 (Y612) and total IRβ, IRS1. β-Actin was used as loading control. ^#^ *p* < 0.05, ^###^ *p* < 0.001, * *p* < 0.05, ** *p* < 0.01 *versus* PA-treated group.

To examine whether PA could impair insulin signal transduction in HepG2 hepatocytes, the effect of PA on insulin-stimulated tyrosine phosphorylation of IRβ, IRS-1/2 and serine phosphorylation of Akt was detected. HepG2 cells were incubated with 0.125 and 0.25 mM PA for 16 h, then the expression level of IRβ, IRS-1/2 and serine phosphorylation of Akt was detected, respectively. The results showed that the insulin-stimulated IRβ tyrosine phosphorylation was significantly inhibited by PA treatment (Figure 4A). IRS-1/2 tyrosine phosphorylation and Akt serine phosphorylation were also blocked by PA. These results show that PA inhibits insulin receptor-mediated signaling pathway in HepG2 cells.

We next investigated whether HPN could reverse PA-induced insulin resistance in HepG2 cells. HepG2 were treated with 0.01, 0.1 and 1.0 μM HPN and 0.25 mM PA for 16 h, and then 100 nM insulin was added into the cells. The results revealed that the IRβ tyrosine phosphorylation was greatly recovered by HPN in a dose-dependent manner (Figure 4B). Tyrosine phosphorylation of IRS-1/2 was also remarkably increased by HPN. At a concentration of 1.0 μM, the expression level of IRS-1/2 almost reached the positive control (insulin-stimulated) group. These results demonstrate that HPN can recover PA-induced insulin resistance in HepG2 cells.

### 2.5. HPN Inhibits PA-Stimulated PTP1B Expression

**Figure 5:**
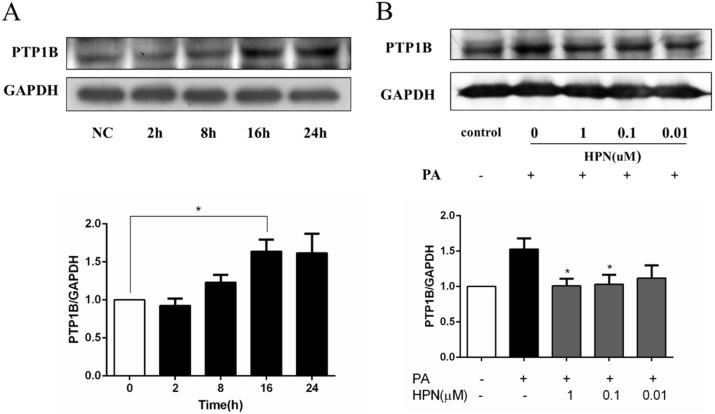
Inhibition of PTP1B by HPN in PA-stimulated HepG2 cells. (**A**) PA up-regulated PTP1B expression. Serum-starved HepG2 cells were untreated or treated with PA (0.25 mM) for 2 h, 8 h, and 16 h. Cells were collected and immunoblotting assay was used to measure the expression of PTP1B. GAPDH was used as loading control. * *p* < 0.05 *versus* non-treated cells. (**B**) HPN blocked PTP1B expression induced by PA in HepG2 cells. HepG2 cells were serum-starved with 0.5% FFA-free BSA medium, and pre-incubated with certain concentration of HPN, followed by treatment with 0.25 mM PA for 16 h. Western blot assay was used to detect the expression changes of PTP1B protein. * *p* < 0.05 *versus* PA-treated group.

We next investigated the expression of PTP1B in PA-treated HepG2 cells. Western blot analysis showed that PTP1B expression level was up-regulated in a time-dependent manner when HepG2 cells were treated with 0.25 mM PA for 2 h, 8 h, and 16 h, respectively (Figure 5A). Interestingly, when HepG2 cells were co-treated with 1.0 μM HPN and 0.25 mM PA for 16 h, PTP1B expression level was decreased to normal levels. Similarly, 0.1 and 0.01 μM HPN could also obviously attenuate PTP1B expression (Figure 5B). The results indicate that HPN improves insulin signaling via blocking PTP1B expression.

### 2.6. HPN Improves Glucose Uptake in HepG2 Cells

**Figure 6:**
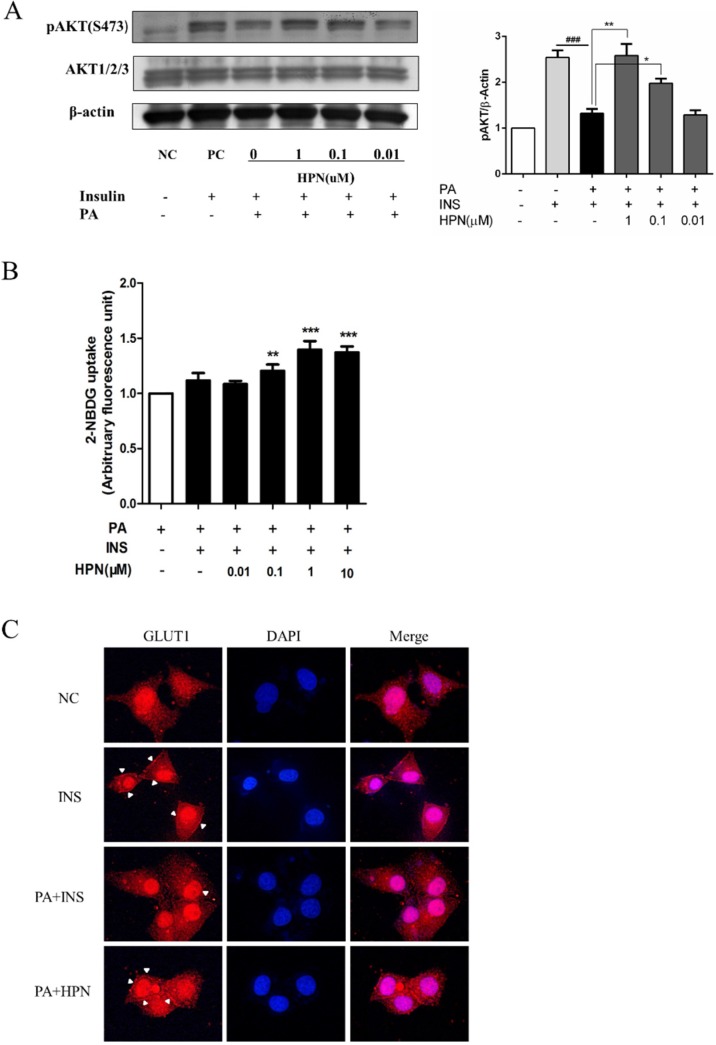
HPN improves glucose uptake ability of HepG2 cells. (**A**) Insulin-stimulated pAkt (S473) was up-regulated during HPN treatment in PA-induced HepG2 cells. HepG2 cells were serum-starved with 0.5% FFA-free BSA medium, and treated with 0.25 mM PA for 16 h after HPN incubation. Subsequently, cells were stimulated with 100 nM insulin for 30 min. Western blot assay was used to determine the changes of pAkt (S473) and total Akt. β-Actin was used as loading control. ^###^ *p* < 0.001, * *p* < 0.05, ** *p* < 0.01 *versus* PA-treated group. (**B**) Microplate fluorimeter measurement of 2-NBDG uptake in PA-treated HepG2 cells. 1 × 10^4^ cells per well were seeded in a black clear-bottomed 96-well culture plate. After overnight incubation, the cells were pre-incubated with HPN for 45 min before PA (0.25 mM) stimulation. After 16 h, cells were cultured in a serum-free low glucose culture medium for 3 h before the addition of 2-NBDG. ** *p* < 0.01, *** *p* < 0.001 *versus* PA-treated group. (**C**) Fluorescent microscopic imaging of Glut1 expression in cell membrane. Cells were treated with indicated concentrations of HPN for 45 min, and incubated for another 16 h with 0.25 mM PA. Then, 100 nM insulin was added. Immunofluorescence assay was described in the Experimental Section.

We further studied the effects of HPN on Akt phosphorylation using antibodies recognizing phospho-serine 473 of Akt. As shown in Figure 6A, cells treated with PA decreased the expression levels of phosphorylated Akt. Interestingly, HPN pre-treatment could significantly recover the activated Akt expression in a dose-dependent manner.

Since Akt phosphorylation can be activated by HPN treatment, we hypothesized that HPN might improve glucose uptake in PA-treated HepG2 cells. To detect whether HPN could ameliorate insulin-stimulated glucose uptake, 2-NBDG, a fluorescent d-glucose analog, was used to monitor glucose uptake in PA-treated HepG2 cells. After HPN and PA treatment, HepG2 cells were incubated with 2-NBDG in a low-glucose DMEM medium for 30 min. HPN-stimulated 2-NBDG uptake level was evaluated by a fluorescent microplate reader. The result showed that HPN could significantly increase glucose uptake potency at concentrations ranging from 0.1 μM to 10 μM. Insulin stimulation could not significantly recover the glucose uptake level compared with PA-treatment cells (Figure 6B).

We next examined whether HPN elevated glucose uptake via the up-regulated Glut1expression in cell membrane. HepG2 cells were treated with 1.0 μM HPN and 0.25 mM PA for 16 h before stimulation with 100 nM insulin, and then the Glut1 expression was determined by immunofluorescence. As shown in Figure 6C, HPN treatment significantly increased the Glut1 levels in the plasma membrane.

The above findings demonstrate that HPN could activate Akt expression and improve glucose uptake in PA-treated HepG2 cells.

## 3. Discussion

Much evidence shows that saturated long-chain fatty acids are the major contributor to lipotoxicity [45]. Palmitic acid is the most common fatty acid exists in mammals. Our studies revealed that PA could induce HepG2 cell death (Figure 2A) and apoptosis (Figure 3C) in a dose-dependent manner. These findings were in line with the previous studies which showed that PA was able to induce apoptotic cell death in various cell types [22,23,24,25]. Recent studies have reported that the expression level of PTB1B may exert a pivotal role in maintaining the balance between survival and death in hepatocytes [18,19,20]. Abnormal increase in PTP1B protein level has been observed in muscle and adipose tissues of diabetic patients [16,17]. Many *in vitro* studies also showed that some factors, such as PA, can up-regulate PTP1B expression and lead to insulin resistance and apoptosis in these cells [31,32,33]. Therefore, therapy strategies based on PTP1B inhibition might be very helpful in diabetic treatment. Our findings demonstrated that HPN, a special PTP1B inhibitor, could significantly protect HepG2 cells against PA-induced cell death (Figure 2C). Using caspase inhibitor as control, the activity of caspase-3 was decreased in a dose-dependent manner (Figure 3B) and the protein ratio of Bcl2/BAX was increased when HepG2 cells were treated with HPN (Figure 3D). These indicate HPN may inhibit PA-induced cell death mainly via apoptosis blocking. All the above findings indicate that HPN can protect hepatocytes from lipid-induced cell death. More importantly, HPN may play an important role in maintaining hepatic cell mass and function in diabetic patients.

Since our previous data have confirmed that HPN has hypoglycemic effects in *db*/*db* mouse model [43], a crucial question of how HPN plays the anti-hyperglycemic role and how HPN attenuates PA-induced insulin resistance still needs to be answered. Our data suggested that HPN suppressed insulin resistance by restoring PA-inhibited insulin signaling in HepG2 cells, including insulin-stimulated tyrosine phosphorylation of IRβ and IRS1/2 (Figure 4B) and serine phosphorylation of Akt (Figure 6A). In addition, FFAs have been reported as a main factor to induce the abnormal expression of PTP1B in rat liver and skeletal muscle cells [46]. Down-regulating of PTP1B leads to enhanced insulin sensitivity [13,14,15]. In the present study, we also found that PA promoted PTP1B expression, while HPN could down-regulate PTP1B protein level in PA-treated HepG2 cells (Figure 5), which further explains how HPN up-regulates insulin signaling. This result also suggests that PTP1B is a modulator of PA-induced insulin resistance and apoptosis.

**Figure 7:**
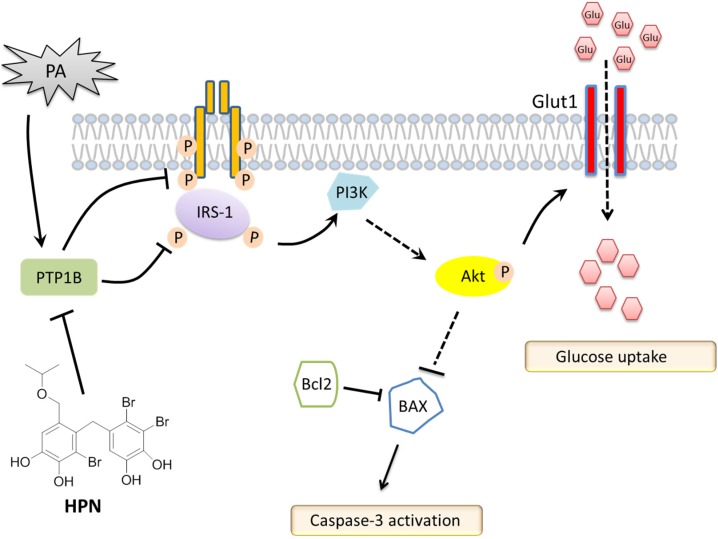
Schematic representation of HPN regulation on hepatic cell death and insulin resistance via PTP1B inhibition. HPN suppressed PA-induced PTP1B expression, which further recovered insulin-stimulated tyrosine phosphorylation of IRβ and IRS1/2. Akt was activated by increased upstream signaling through IR and IRS, thereby regulating PA-induced cell apoptosis, and improved cellular glucose uptake.

Akt, also known as protein kinase B, is a serine/threonine kinase phosphorylated by PI3K. Once Akt is phosphorylated and activated, many genes such as BAD and GSK3 are subsequently regulated. Thus, Akt regulates various cellular processes involved in cell survival and glucose metabolism [6,7]. In this study, we have observed that HPN treatment could significantly recover PA-inhibited phosphorylation of Akt protein level in HepG2 cells (Figure 6A). Further study showed that HPN significantly facilitated glucose uptake in PA-treated HepG2 cells (Figure 6B) and improved the expression of Glut1 in cell membrane (Figure 6C). Therefore, it is likely that HPN activates Akt by increasing insulin signaling through insulin receptor (IR) and insulin receptor substrates (IRS), thereby regulating PA-induced cell apoptosis and reducing glucose uptake (Figure 7).

In summary, the marine bromophenol derivative, HPN, has the ability to prevent human HepG2 cells from PA-induced apoptosis and improves PA-induced insulin resistance. In addition, HPN can also increase HepG2 cell glucose uptake. Since HPN could protect hepatic cell mass and function, it has the potential to be developed as an anti-diabetic agent.

## 4. Experimental Section

### 4.1. Materials

HPN is a synthetic analogue of BPN, which was previously isolated from the marine red alga *Rhodomela confervoides* in our laboratory [43]. HepG2 cells were obtained from Qingdao Medical University Affiliated Hospital (Qingdao, Shandong, China) and cultured in Dulbecco’s modified Eagle’s medium (DMEM) containing 10% (v/v) fetal bovine serum (FBS), penicillin G (100 U/mL) and streptomycin (100 mg/mL) which were purchased from Invitrogen (Carlsbad, CA, USA). Palmitate, insulin and FFA-free BSA were purchased from Sigma-Aldrich (St. Louis, MO, USA). IRS1 antibody, anti-IRS-1 (pS307), Insulin Receptor β (L55B10) Mouse mAb, anti-phospho-IRβ (Tyr1146) rabbit IgG, phospho-Akt (Ser473) (D9E) XP Rabbit mAb, Caspse-3 antibody, Caspse-9 antibody, Bcl-2 (50E3) and Rabbit mAb were obtained from Cell Signaling Technology (Danvers, MA, USA). PTP1B antibody, p-IRS-1/2 Antibody (Tyr 612)-R, anti-β-actin mouse monoclonal IgG (AC-15) and all secondary antibodies were obtained from Santa Cruz Biotechnology (Dallas, TX, USA).GAPDH antibody was purchased from Abclonal Technology (Cambridge, MA, USA). BAX monoclonal Antibody and GLUT1 antibody were obtained from Proteintech Group (Chicago, IL, USA).

### 4.2. Preparation of BSA-Conjugated Palmitate Complexes

Palmitate solutions were prepared as described previously with some modification [22]. Briefly, 100 mM palmitate stock solutions were prepared in 0.1 N NaOH at 70 °C for 10 min with gently shake and then filtered. 5% (wt/vol) FFA-free BSA solution was prepared in double-distilled H_2_O and filtered. 5 mM PA/BSA conjugates were prepared by mixing PA stock solutions with 5% BSA and subsequently incubated at 55 °C for 10 min, then cooled to room temperature, aliquoted and frozen for future use.

### 4.3. MTT Assay

Briefly, HepG2 cells were plated in 96-well plates (5 × 10^3^ cells/well) and incubated overnight. At about 60% confluence, the cells were pre-incubated with HPN (1.0, 0.1 and 0.01 μM) for 45 min before PA (0.5 mM) stimulation. After 24 h, the viability of HepG2 cells was assessed using MTT assay. 10 μL MTT solution (5 mg/mL) was added into each well and the plates were incubated for 4 h at 37 °C, then the MTT-containing medium was removed and replaced with 150 μL DMSO. The absorbance at 490 nm was then measured with an ELx808 microplate reader (BioTek, Winooski, VT, USA). The viability rate of the treated cells was calculated by the formula: cell viability rate (%) = [(A490 sample)/(A490 control)] × 100%.

### 4.4. Caspase-3 Activity Assay

Caspase-3 activity was measured using the Caspase 3 Activity Assay Kit (Beyotime, Shanghai, China) according to the manufacturer’s instructions. Briefly, HepG2 cells were plated in 6-well plates (2 × 10^5^ cells/well). At about 60% confluence, cells were pre-incubated with HPN (1.0, 0.1 and 0.01 μM) for 45 min before PA (0.5 mM) stimulation for 8 h. Cells were subsequently collected and lysed with Lysis Buffer. After Ac-DEVD-pNA (2 mM) addition, the absorbance at 405 nm was measured with an ELx808 microplate reader (BioTek, Winooski, VT, USA). The amount of pNA was then calculated by an according standard curve.

### 4.5. Hoechst 33258 Staining Assay

HepG2 cells were plated in 24-well plates (2 × 10^4^ cells/well), and after overnight incubation, cells were pre-incubated with HPN (1.0, 0.1 and 0.01 μM) for 45 min before PA (0.5 mM) stimulation. After 12 h, cells were fixed with 4% PFA for 10 min and then incubated with 200 uL Hoechst 33258 (10 μM) at room temperature for 30 min. After three washes, cells were observed under the fluorescence microscope (Zeiss, Jena, Germany).

### 4.6. Western Blot Analysis

Western Blot analysis was performed as described previously [47]. Cells were lysed with ice-cold RIPA buffer (Solaibo, Beijing, China). Protein concentration was determined using BCA Protein Assay Kit (Thermo Scientific, Waltham, MA, USA). Cell lysates were separated by SDS-PAGE and transferred onto a NC membrane (Millipore, Billerica, MA, USA). The membranes were blocked with blocking solution (5% nonfat milk/TBST) for1 h at room temperature and subsequently incubated with the following primary antibodies: anti-IRS1, anti-IRS-1 (pS307), anti-IR, anti-p-IRβ (Tyr1146), anti-p-Akt (Ser473), anti-caspase-3, anti-caspase-9 and anti-Bcl2 (1:1000 dilution, Cell Signaling Technology), anti-PTP1B, anti-p-IRS-1/2 (Tyr 612) and anti-β actin (1:1000 dilution, Santa Cruz Biotechnology, Santa Cruz, CA, USA), anti-GAPDH(1:2000 dilution, Abclonal, Cambridge, MA, USA), anti-BAX (1:2000 dilution, Proteintech, Chicago, IL, USA ). After three 5 min washes with TBST buffer, membranes were incubated with rabbit anti-mouse or goat anti-rabbit or rabbit anti-goat HRP-conjugated secondary antibody (Santa Cruz Biotechnology, Dallas, TX, USA) for 1 h. Finally, the chemiluminescence method was employed to detect the signals using Pierce™ ECL Western Blotting Substrate (Thermo Scientific, Waltham, MA, USA) and protein bands were visualized by autoradiography and the intensities were analyzed by Quantity One (Bio-Rad Laboratories, Hercules, CA, USA).

### 4.7. Glucose Uptake Assay

The glucose uptake rate was measured using a fluorescent D-glucose derivative, 2-NBDG, according to the published procedure [48] with some modification. For fluorescent microplate reader measurement of 2-NBDG uptake level, 1 × 10^4^ cells per well were seeded in a black clear-bottomed 96-well culture plate (Corning, NY, USA). After overnight incubation, the cells were pre-incubated with HPN for 45 min before PA (0.25 mM) stimulation. After 24 h, cells were cultured in serum-free low-glucose (5.5 mM) culture medium for 3 h before the addition of insulin and 2-NBDG. 2-NBDG uptake was measured by using a fluorescent microplate reader (BioTek, Winooski, VT, USA). At last MTT assay was performed to adjust the errors generated from cell number difference.

### 4.8. Immunofluorescence

HepG2 cells were plated on glass coverslips, serum starved overnight, treated with HPN (1.0 μM) for 45 min, and then stimulated with PA (0.25 mM) for 16 h. Cells were fixed with 4% PFA for 15 min, blocked with 5% BSA/PBS/Tween20 (0.05%) for 30 min, incubated with GLUT1 antibody (Proteintech, Chicago, IL, USA) for 1 h and then secondary antibody (anti-rabbit cy3) for 1 h. Images were captured with a fluorescence microscope (Zeiss, Jena, Germany).

### 4.9. Statistical Analysis

All the experiments were performed at least three times, and the data are presented as means ± SD values. Differences between the mean values were assessed using one-way analysis of variance. For all the analyses, *p* < 0.05 was considered significant. Statistical analyses were performed using SPSS 17.0 (SPSS Inc., Chicago, IL, USA).

## 5. Conclusions

In our present study, we found that HPN was able to alleviate PA-stimulated hepatic cell damage through apoptosis blocking. Furthermore, HPN could also improve insulin sensitivity with upregulated insulin signal transduction via PTP1B inhibition. Finally, HPN acted as a novel glucose uptake promotor which significantly increases glucose uptake. In light of the critical effects of HPN on inhibition of pro-diabetic milieu-induced hepatocytes apoptosis and improvement of insulin resistance, HPN could be used as a potential drug for T2DM treatment or as a lead compound for the design of other novel therapeutic drugs for type 2 diabetes which protects both hepatic cell mass and function.
